# Protocol for the ROSE sustainment (ROSES) study, a sequential multiple assignment randomized trial to determine the minimum necessary intervention to maintain a postpartum depression prevention program in prenatal clinics serving low-income women

**DOI:** 10.1186/s13012-018-0807-9

**Published:** 2018-08-22

**Authors:** Jennifer E. Johnson, Shannon Wiltsey-Stirman, Alla Sikorskii, Ted Miller, Amanda King, Jennifer L. Blume, Xuan Pham, Tiffany A. Moore Simas, Ellen Poleshuck, Rebecca Weinberg, Caron Zlotnick

**Affiliations:** 1 Division of Public Health, Michigan State University, 200 East 1st St Room 366, Flint, MI 48502 USA; 2 National Center for PTSD, Dissemination and Training Division and Stanford University Department of Psychiatry and Behavioral Sciences, 795 Willow Road (NC-PTSD 334), Menlo Park, CA 94025 USA; 3 Department of Psychiatry, Michigan State University, 909 Fee Rd Room 321, East Lansing, MI 48824 USA; 4 Pacific Institute for Research and Evaluation, 11720 Beltsville Drive Suite 900, Calverton, MD 20705 USA; 5 Mayo Clinic, 200 First St, Rochester, MN 55901 USA; 6 Michigan Medicine, 1500 E. Medical Center Drive, Ann Arbor, MI 48109 USA; 7 Spectrum Health, 221 Michigan Street, Suite 402 MC 118, Grand Rapids, 49503 USA; 8 University of Massachusetts Medical School/UMass Memorial Health Care, Memorial campus - 119 Belmont Street - Jaquith 2.008, Worcester, MA 01605 USA; 9 University of Rochester Medical Center, 601 Elmwood Ave, Box PSYCH, Rochester, NY 14642 USA; 10 Allegheny Health Network, 4815 Liberty Avenue, Mellon Pavilion, Suite GR4, Pittsburgh, PA 15224 USA; 11 Butler Hospital and Brown University, 345 Blackstone Blvd, Providence, RI 02906 USA; 12 University of Cape Town, Cape Town, South Africa

**Keywords:** Implementation, Sustainment, Cost-effectiveness, Postpartum depression, Prevention, Public assistance, Prenatal care

## Abstract

**Background:**

More research on sustainment of interventions is needed, especially return on investment (ROI) studies to determine cost-benefit trade-offs for effort required to sustain and how much is gained when effective programs are sustained. The ROSE sustainment (ROSES) study uses a sequential multiple assignment randomized (SMART) design to evaluate the effectiveness and cost-effectiveness of a stepwise approach to sustainment of the ROSE postpartum depression prevention program in 90 outpatient clinics providing prenatal care to pregnant women on public assistance. Postpartum depression (PPD) is common and can have lasting consequences. Outpatient clinics offering prenatal care are an opportune place to provide PPD prevention because most women visit while pregnant. The ROSE (Reach Out, Stay Strong, Essentials for mothers of newborns) program is a group educational intervention to prevent PPD, delivered during pregnancy. ROSE has been found to reduce cases of PPD in community prenatal settings serving low-income pregnant women.

**Methods:**

All 90 prenatal clinics will receive enhanced implementation as usual (EIAU; initial training + tools for sustainment). At the first time at which a clinic is determined to be at risk for failure to sustain (i.e., at 3, 6, 9, 12, and 15 months), that clinic will be randomized to receive either (1) no additional implementation support (i.e., EIAU only), or (2) low-intensity coaching and feedback (LICF). If clinics receiving LICF are still at risk at subsequent assessments, they will be randomized to either (1) EIAU + LICF only, or (2) high-intensity coaching and feedback (HICF). Additional follow-up interviews will occur at 18, 24, and 30 months, but no implementation intervention will occur after 18 months. Outcomes include (1) percent sustainment of core program elements at each time point, (2) health impact (PPD rates over time at each clinic) and reach, and (3) ROI (costs and cost-effectiveness) of each sustainment step. Hypothesized mechanisms include sustainment of capacity to deliver core elements and engagement/ownership.

**Discussion:**

This study is the first randomized trial evaluating the ROI of a stepped approach to sustainment, a critical unanswered question in implementation science. It will also advance knowledge of implementation mechanisms and clinical care for an at-risk population.

**Trial registration:**

Clinicaltrials.gov, NCT03267563. Registered June 14, 2018.

## Background

More research on sustainment of interventions is needed. A recent expert consensus report concluded that, “Little is known about how well or under what conditions health innovations are sustained and their gains maintained once they are put into practice” [1]. This report placed high priority on conducting return on investment (ROI) studies to determine how much is gained when effective programs are sustained and cost-benefit trade-offs for effort required to sustain [1]. Limited empirical information on methods and benefits of sustainment can result in (1) discontinuation despite significant investment in initial implementation, or in (2) policymakers being unsure about whether resources should be devoted to implementation and scale-up [1]. The ROSE Sustainment (ROSES) Study evaluates the effectiveness and cost-effectiveness of a stepwise approach to sustainment of an evidence-based postpartum depression prevention program in 90 outpatient prenatal clinics serving women on public assistance.

### Clinical context

Postpartum depression (PPD) is a common and impactful public health problem, especially among low-income women. A meta-analysis derived an average prevalence rate of 13% for PPD within the first 12 weeks postpartum [2]. Untreated PPD can have severe and lasting consequences for mother and infant [3–10].

Low-income women have higher rates of PPD (up to 50%) [11–15] than do other income groups [16, 17], are less likely to receive treatment for their PPD [18, 19], and have more severe consequences of untreated PPD on maternal caregiving and child development [7, 20, 21]. Therefore, timely and effective interventions to reduce their PPD risk (i.e., to prevent PPD rather than treat a full blown episode) are critical. However, health professionals have remained focused on identifying and treating perinatal depression after its onset [22, 23], rather than preventing it. Outpatient clinics offering prenatal care are an opportune place to provide PPD prevention because most women visit while pregnant.

The ROSE (Reach Out, Stay Strong, Essentials for mothers of newborns) program is an evidence-based practice for reducing cases of PPD among low-income and racially and ethnically diverse women [24]. Designed to address the high risk of PPD among low-income women, ROSE is administered to pregnant women in small groups, at outpatient clinics providing prenatal care. ROSE teaches interpersonal psychotherapy (IPT)-based skills for improving communication and building social support, identified risk factors for PPD. [2, 25, 26] ROSE is presented as a course to minimize stigma and emphasize the program as an educational experience. ROSE consists of four 90-min group sessions and a post-delivery 50-min individual booster session, with easy to read handouts and homework for each session. Two fully-powered randomized trials [27, 28] and a randomized pilot trial [29] support its effectiveness in reducing cases of PPD among low-income women in the first 3–6 months following childbirth (Table 1).

**Table 1:** Randomized trials examining the effectiveness of ROSE in preventing PPD

Population	Sample size	% with PPD: ROSE	% with PPD: usual care	Time post-partum
Pregnant women on public assistance [29]	37	0%*	33%	12 weeks
Women on public assistance at risk for PPD [27]^a^	99	4%*	20%	3 months
Pregnant women on public assistance at PPD risk [28]	205	16%*	31%	6 months
Pregnant adolescents [39]	106	12.5%	25%^b^	6 months
African-American women at risk for PPD [40]^a^	36	Depressive symptoms decreased over time	No change in depressive symptoms	3 months

*p < .05 between conditions

^a^Per Cooper Survey Questionnaire [76]

^b^Dose-matched control

ROSE is flexible, easy to implement, and can be delivered by individuals already working in prenatal clinics. The ROSE manual is highly scripted. Nurses or medical assistants can deliver ROSE. ROSE overcomes barriers to attendance for low-income women by coordinating sessions with women’s prenatal clinic appointments and having a flexible delivery structure (see Table 2).

**Table 2:** Example ROSE core elements and adaptable periphery

Examples of ROSE core elements	Adaptable periphery
Psychoeducation on: • PPD • Managing stress in transition to motherhood • Social support as a buffer against PPD • Relevant postpartum resources Teaching: • Communication skills via role plays • Stress management skills • Building and enhancing social skills Review/reinforcement of skills at postpartum session	Group vs. individual Office vs. home visit Time during pregnancy Order of sessions Open enrollment of group Missed sessions can be made up Sessions can be split into shorter pieces or lumped together

### Implementation and sustainment framework and rationale

#### Conceptual framework guiding choice of assessments

According to reviews [30–32], a program is sustained where there is a continuation of its core elements at sufficient fidelity, continuation of intended health benefits (i.e., prevention of PPD), and adequate capacity for continuation of core elements is maintained. Capacity is “the extent to which a community has local access to the knowledge, skills, and resources needed to conduct the program effectively” [32]. This definition of sustainment and the RE-AIM framework (reach, effectiveness, adoption, implementation, and maintenance) [33] provide the conceptual framework guiding assessments for this study. Study outcomes include reach (number of patients receiving and completing ROSE), effectiveness (PPD rates over time), and adoption (time from initial training to offering program). Implementation consistency (i.e., fidelity to core program elements) over time is a primary outcome; implementation costs and processes (e.g., adaptations, barriers) are secondary outcomes. Our other primary outcome is maintenance (months ROSE is provided with adequate fidelity). One hypothesized mechanism (clinical and organizational capacity) is also derived from our definition of sustainment. The other hypothesized mechanism (engagement/ownership) was proposed by Shediac-Rizkallah and Bone to be an important facilitator of sustained capacity (they suggest that participation ➔ownership ➔ sustained capacity ➔sustained program) [32]. The need for research determining the ROI of sustainment comes from an expert consensus research agenda on sustainment [34]. The need for an examination of processes and our chosen predictors (organizational and policy contexts) are clearly articulated by all these authors [30, 32, 34].

#### Implementation interventions being tested in current study and rationale

Choice of implementation interventions for the ROSE Sustainment (ROSES) Study is based on the replicating effective programs (REP) framework [35]. For implementing ROSE in prenatal clinics, all the implementation pre-conditions of REP have been met. We have identified a high-burden condition (PPD), identified an effective intervention that fits prenatal clinics (ROSE), and packaged the intervention (i.e., it has already been packaged for, tested with, and found feasible, acceptable, and effective using prenatal clinic nurse health educators as interventionists [27–29]).

**Fig. 1:**
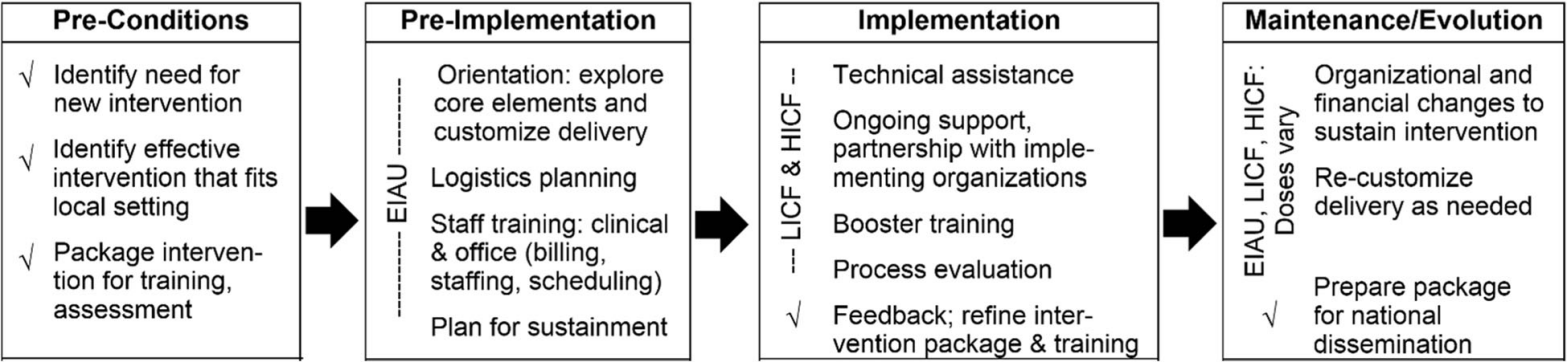
Study implementation interventions (EIAU, LICF, HICF) fit the replicating effective programs (REP) framework. Detailed legend: Figure adapted from Kilbourne et al. [35]

Implementation and sustainment interventions used in the current study are guided by the next three REP phases. Enhanced implementation as usual (EIAU; initial training + tools for sustainment) will consist of the pre-implementation steps shown in the framework (see Fig. 1). Initial training will include an explanation of the core elements, discussion about how delivery can (and should not) be customized (see Table 2), logistics planning, and staff training (including training of clinical staff and work with office staff to clarify billing, scheduling, and staffing). The two experimental sustainment conditions (i.e., low-intensity coaching and feedback [LICF] and high-intensity coaching and feedback [HICF]) will contain lower (every 3 months) and higher (every month) doses of REP’s implementation steps (technical assistance, ongoing support of and conversation with the clinics, coaching [including booster training and process evaluation], feedback; Fig. 1). The three implementation interventions provide varying doses of guidance for re-customizing delivery and making organizational and financial changes to sustain the intervention, as suggested in the framework’s maintenance and evolution phase.

### ROSE Sustainment (ROSES) Study aims

The ROSES Study evaluates cost-effectiveness of a stepwise approach to sustainment of ROSE in 90 outpatient prenatal clinics serving women on public assistance. Specific aims are to

1. Compare effectiveness of each sustainment step for the following final and proximal outcomes:
  - Percent sustainment of core program elements at each time point (primary) and total length of time that (i) any ROSE services were provided and (ii) were provided with adequate fidelity to core elements.
  - Health impact (e.g., PPD rates over time at each clinic) and reach (number of patients enrolled in and completing the ROSE program).
  - Return on investment (costs and cost-effectiveness of each sustainment step)
  - Hypothesized mechanisms including sustainment of (i) clinical and organizational capacity to deliver core elements, and (ii) a sense of engagement/ownership by key clinic staff
2. Examine predictors and processes to determine which kinds of clinics need which level of support:
  - Explore which clinic characteristics (e.g., organizational and state policy contexts) and hypothesized mechanisms (Aim 1d) are associated with best sustainment to determine tailoring variables for choosing/sequencing EIAU, LICF, and HICF in the future.
  - Document implementation/sustainment processes, their timing relative to desired outcomes, and critical incidents to explore factors most related to sustainment after accounting for hypothesized mechanisms.

### Innovation

The ROSES Study is innovative in that there are few randomized implementation studies with sustainment as primary outcome. In particular, identifying the “minimum necessarily to sustain” is a novel methodological [36] aspect of this study. In addition, the ROSES Study is the first implementation study of a PPD prevention intervention in outpatient clinics providing prenatal care. In fact, virtually no interventions that prevent any mental health problem among adults without requiring the use of mental health clinicians have been the subject of implementation research in outpatient medical settings [37].

Finally, dynamic tailoring of implementation interventions is needed to enhance the science of implementation. Clinic and contextual factors lead to heterogeneity in response to implementation interventions. The study’s sequential multiple assignment randomized trial (SMART) design, in which we adjust intervention intensity based on risk of failure to sustain, addresses this heterogeneity. Results will build a needed evidence base for precision algorithms for optimizing the allocation of implementation resources to achieve sustainment.

## Methods

### Preliminary studies

#### Past work on ROSE

ROSE is effective at reducing PPD cases among low-income women in community settings (see Table 1). Given the highly scripted intervention manual, interventionists with varying qualifications (i.e., paraprofessionals, nurses) have delivered ROSE with fidelity [38]. Delivering ROSE in prenatal clinics is feasible and acceptable to low-income pregnant women, with good session attendance [39, 40] and high perceived helpfulness of intervention components [27]. ROSE is also feasible and acceptable to clinics serving low-income women, as demonstrated by requests for the ROSE manual and/or training across the USA and in Japan, as well as surveys conducted in Michigan (see below).

#### Pilot work in Michigan

Included staff surveys in 13 prenatal clinics (*n* = 27 respondents). Data suggested that clinics were motivated to implement ROSE. All respondents viewed preventing PPD as “very important” (*n* = 24) or “important” (*n* = 3). All but one respondent was “somewhat” to “very” interested in implementing ROSE for their patients. Data also suggested that clinics had a need for ROSE. As is the case nationally, none of the surveyed clinics had strategies in place to prevent PPD. Instead, most clinics (11/13) tried to provide some regular screening for PPD after birth, indicating that PPD is a priority issue. Data also suggested that implementing ROSE in prenatal clinics is feasible. Ten clinics immediately identified staff that had adequate time and training to lead ROSE groups; three were unsure. EIAU, LICF, and HICF will all work with clinics to identify ROSE group leaders and manage workflow.

When asked what about ROSE might be a good fit for their clinics, respondents described a need (“our patients have significant modifiable risk factors,” “at least half of the pregnant patients are presenting with depression and/or anxiety”), and said that it is better to prevent PPD than to deal with it after it occurs. They also mentioned the non-threatening nature of ROSE (which does not require women to endorse PPD) and the convenience of coming to a familiar office. Implementation facilitators included “We have numerous patients; low income, minority, history of depression,” “nursing staff well aware of the social problems our patients face,” “group setting would increase patients being seen and increase comfort,”and “clients more apt to attend visits.” Using questions based on Steckler [41], respondents agreed that ROSE would be more effective in preventing PPD than current practices and would improve quality of care at their clinics and disagreed that it would be difficult to learn. Motivation of staff to address PPD was viewed as a facilitator.

Clinics were also able to identify supports needed to implement and sustain ROSE, which we integrated into our study implementation conditions. On a scale from “1 = serious barrier” to “7 = strong facilitator,” staff training (4.2), staff time and workload (3.4), billing issues (3.5), and space (3.3) were viewed as neutral to slight barriers, but none was seen as a serious barrier. Informed by these responses, training, time and workload, billing, and space issues are addressed in varying degrees in the three study conditions (EIAU, LICF, and HICF). Respondents rated components of EIAU, LICF, and HICF as important for successful implementation of ROSE at their clinics (see Table 3).

**Table 3:** Pilot data: Clinic ratings of need for implementation supports

Ratings of need for implementation supports (1 = least needed, 10 = most needed)	
Supports provided once in EIAU and on an ongoing basis, in higher doses, and in LICF and HICF	Materials for patients about why they might want to participate (9.38)
	Initial training (8.85)
	Someone to talk with staff about why the program matters (8.62)
	Work with office to figure out how to pay for (8.42)
	Educate staff about PPD/mental health (8.12)
Supports provided only in LICF and HICF:	Audit and feedback (to show that ROSE is working and how; 8.38)
	Ongoing training (7.50)

### Critical design decisions

#### Rationale for implementation interventions and a SMART design

The goal of the ROSES study is to determine the minimum intervention needed to sustain ROSE in clinics that provide prenatal services to women on public assistance and the optimal timing of boosters. To achieve this goal, the implementation interventions (EIAU, LICF, and HICF) were chosen to reflect different intensities of a standard approach to allow their intensity/cost aspects to take precedence. When an intervention does not produce a desired outcome, two options are available: to give it more time or step up the intervention intensity. The study’s SMART design allows us to isolate the effect of intensifying an intervention (i.e., stepping up to LICF or HICF) versus giving a simpler one (such as EIAU or LICF) more time. It also builds an evidence base for precision algorithms optimizing the allocation of implementation resources to achieve sustainment.

#### Rationale for sample

We will enroll 90 outpatient medical clinics providing prenatal care in Michigan, Rhode Island, Massachusetts, Pennsylvania, New York, and Florida. We chose to include any kind of outpatient clinic for which ROSE would be appropriate (e.g., federally qualified heath centers [FQHCs], hospital-affiliated clinics, independent practices, and visiting nurses) in order to speed knowledge acquisition relevant to widespread scale-up. To be included in the study, clinics will be (1) outpatient, (2) provide prenatal services, (3) estimate that at least 50% of their pregnant patients receive some kind of public assistance (such as federal or state cash assistance, food stamps, subsidized housing, and/or health insurance such as Medicaid), (4) have at least ten new pregnant women per month on average (i.e., enough patient flow to run ROSE), and (5) agree to study procedures. Given the need for prevention of PPD among low-income women, the study addresses ROSE implementation to clinics serving mainly low income women; however, any woman within the clinic can receive ROSE.

### Research design

#### Implementation interventions

Except for one in-person training in HICF, meetings and trainings take place by videoconference or telephone. Trainings and meetings for each clinic will be recorded and provided for optional later viewing.

EIAU consists of initial training and problem-solving plus planning for sustainment and covers the pre-implementation step of the REP framework (Fig. 1). Step 1 study investigators will meet with key clinical and operational staff. This 2-h meeting will include (1) a brief clinical and operational overview of ROSE, (2) problem-solving and discussion around adaptable elements of ROSE, and (3) planning and tools for sustainment. This collaborative process will mesh the clinic’s context, needs (including needs of patient population), and resources, with discussion of ROSE core and adaptable elements, resulting in a written, tailored implementation and sustainment plan that identifies who within the clinic is responsible for what. Step 2 will consist of two separate video meetings. The first videoconference (60–90 min) will include operational staff to discuss operational issues (such as reimbursement, identification and referral procedures, and identification of suitable providers). The second videoconference will consist of a live 4-h training for providers on how to conduct ROSE. Providers will be given a manual with the ROSE program, patient handouts, a summary of key components, scripts for presenting ROSE to patients, and a customized description of the clinic’s logistics for ROSE. Because training sessions are recorded and there is a written, clinic-specific sustainment plan, it is possible for clinics to replenish staff turnover, but turnover may create risk of not sustaining, which would be addressed in LICF or HICF.

LICF will include EIAU plus low-intensity coaching and feedback, consisting of three components. The first component includes one clinical and one operational telephone “booster” meeting quarterly for additional support (up to 1 h each). Meetings will identify challenges to conducting ROSE with fidelity, collaboratively problem solve solutions, discuss re-customization of delivery if needed, and develop an action plan to address barriers. Subsequent meetings will review implementation progress and collaboratively make changes to the action plan based on new data, experiences, and discussion.

The second component of LICF is provision of feedback to clinical and operational staff during booster meetings to help guide discussion and planning. Clinical feedback will include information about their fidelity to core ROSE elements based on the ROSE session-by-session adherence scale and interview validation (see primary outcome section). We will also provide information to clinical and operational staff on any changes in the clinic’s rates of PPD for the previous quarter and on challenges or successes we detect from survey measures.

Finally, to promote partnership and ownership, clinical and operational staff from clinics assigned to LICF will be invited to participate quarterly in collaborative board phone meetings with study investigators and staff from other study clinics. Staff from LICF clinics will provide feedback to the study team about the implementation strategies being used, helpful adaptations to the intervention that preserve core elements, and ways to address challenges in other clinics.

HICF: Clinics in the HICF condition will receive everything that the clinics in LICF receive, but at a higher intensity. Clinical and operational booster meetings, feedback, and participation in collaborative board meetings will be monthly, rather than quarterly. In the month after randomization to HICF, study investigators will travel to the clinic to provide an in-person clinical and administrative “booster” meeting to increase engagement (a proposed mechanism). The remaining monthly meetings will be by telephone or videoconference. Study investigators will also be available to answer questions on an ad-hoc basis.

#### Characterizing implementation interventions (EIAU, LICF, HICF)

Every implementation encounter (e.g., initial or ongoing training, collaborative board meetings) will be documented in an electronic implementation case note and audio or video recorded. The case note will include encounter length, time spent on operational vs. clinical support, a checklist of implementation strategies used (taken from Powell et al., 2015) [42], a checklist of discussion topics (e.g., billing options), and free response sections to describe clinic staff responses. We will review and rate 20% of the recordings to verify/augment the notes.

#### Randomization

**Fig. 2:**
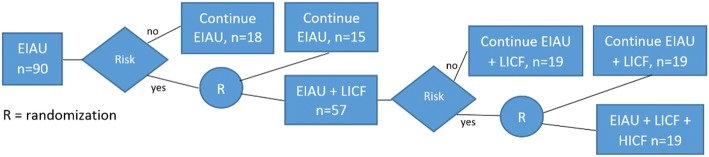
The ROSE Sustainment (ROSES) Study SMART design

After the baseline assessment, all clinics will receive EIAU (initial training + tools for sustainment). Clinics that are determined to be at-risk for operational (defined as no ROSE intervention in 3 months and none planned) and/or clinical (defined as less than 75% fidelity to ROSE core elements) failure to sustain at subsequent assessments up to 15 months will be randomized to receive additional support. At the first time period at which a clinic is determined to be at risk (i.e., at 3, 6, 9, 12, or 15 months), that clinic will be randomized in a 3.8:1 ratio (Fig. 2) to receive either (1) the addition of low-intensity (every 3 months) coaching and feedback (LICF), or (2) no additional implementation support (EIAU only). If clinics receiving LICF are still found to be at risk at subsequent monitoring periods, they will be randomized in a 1:1 ratio to either (1) nothing additional (i.e., EIAU + LICF only), or (2) high-intensity (monthly) coaching and feedback (HICF). Additional study follow-up interviews will occur at 18, 24, and 30 months, but no implementation intervention will occur after 18 months. Randomization procedures will balance trial arms by time (3, 6, 9, 12, or 15 months) and whether or not the clinic is a FQHC. Figure 2 shows the SMART design.

Statistical power is based on the primary outcome: percent sustainment of core ROSE elements at each time point. We start with powering the comparison created by the second randomization, EIAU+LICF vs. EIAU+LICF+HICF. For this comparison, the literature reports a range of effect sizes, with three out of four most relevant ones [43–45] exceeding Cohen’s *d* = 0.48. Aim 1 analyses include adjustment for the 6-month version on the primary outcome (baseline for second randomization) and repeated measures at 9, 12, 15, 18, 24, and 30 months. Assuming correlations between pairs of repeated measures of 0.7 based on past work, *n* = 19 per group is needed to detect the target effect size with power of 0.80 or greater at *α* = 0.05 in two-tailed tests. Assuming that two thirds of the clinics would still be at risk after EIAU + LICF, *n* = 38 clinics will enter into the second randomization. Therefore, one third, or *n* = 19 clinics would be deemed low risk and continue EIAU+LICF. Moving to the left in Fig. 2 to the first randomization, the EIAU + LICF group will have size *n* = 57 as determined above. The size of the other group in the first randomization of *n* = 15 will allow to detect the target effect size with power of 0.89. After the initial EIAU period, we expect approximately 80% of clinics to be at risk [45, 46]. Therefore, 72 (57 + 15) clinics in the first randomization will be 80% of the sample. Thus the initial sample size receiving EIAU will be *n* = 90.

Assessments will be conducted at 0, 3, 6, 9, 12, 15, 18, 24, and 30 months. Each clinic will identify two people: the person most involved in ROSE (1) clinical and (2) operational (e.g., billing and scheduling) functions. We anticipate two respondents per clinic, but power and measures are unaffected if they are the same person. Clinic-level measures will be derived from the surveys and analyses occur at the clinic level. Respondents will complete quantitative measures online. Qualitative interviews will take place by phone (see Table 4).

**Table 4:** ROSES Study schedule of assessments

Assessments	Type	Respondent			Base-line	Follow-ups
		opera-tional	clinical	records*		
Sustainment (primary and secondary outcomes)						
Core elements (delivered/should have been delivered over 3 months)	Session **√**list			X		X
Months ROSE offered, months offered with fidelity	Interview, √list		X	X		X
Health impact (# screened, # screened positive for PPD at clinic)	Objective			X	X	X
Reach (# pts. attending ROSE, # completing ROSE)	Objective			X		X
Mechanism: capacity						
Program assessment Sustainability tool: organizational capacity	Survey	X	X		X	X
# trained, trained w/time to deliver, trained and done w/fidelity	Survey, **√**list		X	X		X
Manage space, able to bill for ROSE?	Survey	X			X	X
Mechanism: ownership/engagement						
Program assessment Sustainability tool: four subscales	Survey	X	X		X	X
NHS Sustainability Model and Guide: staff section	Survey	X	X		X	X
Attitudes toward PPD	Survey	X	X		X	X
ROI: costs	Objective			X		X
Predictors and processes						
Implementation climate assessment	Survey	X	X		X	X
State policy context: legislation, maternal mortality rates	Objective				X	X
Dates, process notes from training, and coaching/feedback sessions	Objective			X		X
Other processes and critical incidents	Qualitative	X	X			X
Clinic descriptives	Survey	X			X	X

*Based on records kept by the clinic or the study. The study will not have direct access to individual medical records

We define operational failure to sustain as no ROSE intervention in 3 months and none planned. We define clinical failure to sustain as less than adequate fidelity to ROSE core elements (i.e., an average of < 75% of core elements for each session delivered, as measured by the ROSE session-by-session adherence scale; see primary outcome section below). Used in previous ROSE trials (Table 1), this checklist lists on average eight items (rating of present/absent) that assess whether key tasks of each session were completed. Given that ROSE is scripted, item answers are in yes/no format, and core elements are basic (e.g., yes/no—did interventionist explain PPD? Did interventionist have group members practice communication skills through role plays?); completing 75% of these for “adequate” fidelity is reasonable.

#### Primary outcome

Our primary outcome reflects the effectiveness of each sustainment step in terms of (a) percent sustainment of core program elements at each time point and (b) total length of time any ROSE services were provided, and length of time they were provided with at least moderate fidelity to core elements. Statistically, one of these measures should be the primary outcome; we have chosen the first because it has repeated measures over time, improving power. ROSE’s core program elements (Table 2) will be assessed through the ROSE session-by-session adherence scale, a self-rated intervention fidelity scale completed by ROSE interventionists after each session [28]. The outcome for each time point (i.e., quarter) will be the mean percent of core elements delivered that should have been delivered at each ROSE session (mean [number of core elements delivered/number of core elements should have been delivered] at each session; zero if no sessions were completed). Self-reported checklists of mental health intervention fidelity have shown excellent validity when compared to observer-rated scales [47–50]. We will validate checklist responses against expert ratings using qualitative interviews for three sessions per quarter. Using a monthly calendar method [51, 52], we will track (1) the total length of time any ROSE services were provided, and (2) total amount of time ROSE services were offered with adequate fidelity of core elements (defined as 75% or more on the ROSE session-by-session adherence scale averaged across interventionists and sessions each month).

#### Secondary outcomes

##### Health impact (PPD rates over time at each clinic)

Quarterly, we will ask each clinic to report the following overall numbers: (1) number of women who should have come for their 6-week postpartum appointment; (2) number who came; (3) number that were screened for PPD; and (4) number who screened positive for PPD. We will also collect this information for four quarters (12 months) prior to baseline. We will use these numbers to calculate PPD rates for each time period (see below). Although not every woman in the clinic will receive ROSE, we chose to track overall PPD rates at each clinic because: (1) the study examines larger-scale sustainment aimed at clinic-wide (and eventually population-wide) outcomes; and (2) the clinic-level outcomes are primary; not consenting individual patients makes the needed sample size (90 clinics) feasible. Number of patients enrolled in and completing ROSE (i.e., reach) [1]. Clinics will track the number of: (1) patients who agree to come to ROSE, (2) patients attending at least one session, and (3) patients attending at least three of the five sessions. Clinic size (number of new pregnant patients per quarter) and estimated percent of clinic patients on public assistance will be considered in analyses.

##### Return on investment

We will analyze four cost-effectiveness outcome measures: (1) a primary clinical outcome and number of PPD cases averted, estimated as the change in PPD rate at the clinic (post-pre)*(clinic’s caseload); (2) another clinical outcome and number of quality-adjusted life years saved, computed from the primary outcome using Morrell et al.’s [24] model; (3) an implementation process outcome and number of clients served with fidelity; and (4) a sustainment outcome and months of additional service delivery. Our grant accounting will capture our costs to provide EIAU, LICF, and HICF. Clinic costs to receive EIAU, LICF, and HICF will be assessed using hours that clinic staff spent on EIAU, LICF, or HICF; associated direct costs (e.g., printing) and staff salaries; and fringe benefits and overheads. In addition to assessing the cost-effectiveness of EIAU, LICF, and HICF, we will also assess the cost-effectiveness of ROSE itself. We will track ROSE delivery costs at each clinic for one pay period (i.e, 2 weeks) using a time sheet for staff who spend time on ROSE to record their ROSE-related hours, work hours on other programs, residual personal overhead hours, and training time.

##### Proposed mechanism 1: Clinical and organizational capacity to deliver ROSE

The primary measure will be the organizational capacity subscale of the Program Sustainability Assessment Tool [38, 53]. Secondary measures include number of people trained who have time to deliver ROSE and perceiving the clinic as able to manage space/scheduling and to bill/get reimbursed for ROSE.

##### Proposed mechanism 2: Ownership and engagement by clinic staff

Ownership and engagement by clinic staff will be assessed using the sum of other relevant subscales of the Program Sustainability Assessment Tool (primary): communications, partnerships, political support, and strategic planning [38, 53]. The Staff section of the National Health Service’s Sustainability Model and Guide [54, 55] and investment in addressing PPD will be secondary measures.

We will assess predictors and processes to provide information about which kinds of sites need which level of sustainment support. Organizational context will be assessed using Aarons’ implementation climate assessment [56, 57]. State policy context will be assessed through two measures: (1) Enacted state legislation about PPD (0 = no state enacted state legislation related to PPD, 1 = awareness-related PPD legislation, 2 = legislation mandating PPD education and services, 3 = legislation with money attached for PPD education/services); and (2) state-level maternal mortality [58, 59].

Processes of implementation and sustainment efforts will be documented using qualitative interviews and implementation case notes. Timing: We will record the dates of EIAU, LICF, and HICF interventions and dates of any change in ROSE status (i.e., ROSE offered, not offered, and ROSE offered with fidelity vs. not) to examine the temporal relationships among these events. Respondent perceptions of critical incidents to sustainment success or failure will be assessed using qualitative interviews.

#### Analyses

Clinic is the unit of randomization and analysis. Primary analyses will be intent-to-treat. All statistical tests will be two-sided with *α* = 0.05 for sustainment of core program elements (primary) and health impact (secondary) outcomes, specified a priori. In exploratory analyses, false discovery rate due to multiple tests will be controlled using the Hochberg adjustment [60, 61].

##### Baseline comparisons, regression techniques, and missingness

Outcome values at baseline, minimization variables, and clinic characteristics will be compared using *t* tests, chi-square test, or Fisher’s exact test. If systematic differences are found, they will be considered as covariates in further analyses. Since analyses will control for baseline values of outcomes, the extent to which other factors may affect post-randomization outcomes will be reflected in the baseline version. The regression techniques described below allow for missing at random mechanisms [62]. If patterns of missing data indicate not missing at random mechanisms, then models describing missing mechanisms will be considered (e.g., pattern-mixture models) [63, 64], and sensitivity analyses will be employed to investigate the robustness of the results.

Specific Aim 1a, hypothesis 1: Among clinics determined to be at risk after EIAU + LICF, those randomized to receive HICF will have better percent sustainment of core program elements at post-randomization assessments than those randomized to continue EIAU + LICF alone. Hypothesis 2: Among clinics at risk after EIAU, those randomized to EIAU + LICF in the first randomization will have better percent sustainment of core program elements at post-randomization time points than those randomized to continue EIAU alone. Hypotheses will be tested using generalized linear mixed effects (GLME) models with seven repeated measures for the first randomization and six for the second. Outcome value at 3 months for the first randomization (after the initial EIAU), and at 6 months for the second one (after the initial EIAU+LICF), will be entered as covariates in the respective analyses. Time will be entered as a class variable to model potentially non-linear patterns. The test of the equality of the coefficient for the randomized condition to zero in the GLME model will yield the test for the main (time-averaged) effect of each step-up intervention. To explore any changing intervention effect as time progresses, time by randomized condition interaction will be added to the model. Specific Aim 1b will use an approach similar to Aim 1a.

Specific Aim 1c: We will use an incremental cost-effectiveness analysis, showing cost-effectiveness ratios for EIAU, for adding LICF, and for adding HICF. The cost-effectiveness ratio equals ΔC/ΔE, where ΔC is the difference in costs as LICF and HICF are added, and ΔE is the difference in the outcome measure. We will bootstrap 95% confidence intervals around the cost-effectiveness ratio and conduct sensitivity analyses.

Specific Aim 1d: Mechanisms of effects of LICF, HICF. Randomized condition will be treated as the independent variable, and hypothesized mechanisms (primary measures of capacity and engagement/ownership) will be treated as potential mediators (one at a time). Effects of mediators on outcomes will be tested by adding them to the GLMEs described above. We will use a bias corrected bootstrapping analytic strategy [65, 66] to estimate confidence intervals around the indirect effect of randomized condition on the outcome, through the mediator. To establish mediation, the 95% confidence interval must not include zero.

Specific Aim 2a explores which kinds of clinics need EIAU, LICF, or HICF and when for optimal sustainment. Characteristics of clinics found to be at risk at each assessment will be compared to those of clinics found to be not at risk using *t* tests, chi-square test, or Fisher’s exact test. Clinic characteristics (size, percent on public assistance, yes/no FQHC), organizational context (implementation climate assessment score), state policy context (rating of state PPD legislation), and hypothesized mechanisms (capacity and engagement/ownership assessed using Program Sustainability Assessment Tool subscale scores) will be considered as potential tailoring variables in defining optimal intervention sequences. The optimal decision rule will be formulated to specify best first and second intervention stage following the initial administration of EIAU. For example, a decision rule might be if a clinic is FQHC, start with LICF and if found to be at risk at 12+ months, step up to HICF, but if found at risk at 6 or 9 months, give LICF more time. The analysis approach follows the optimization method called Q-learning [67–70] with backward induction [71–73].

Specific Aim 2b examines implementation processes, their timing relative to desired outcomes, and critical incidents in order to explore additional factors related to sustainment, as well as dose-response and active ingredients. For quantitative analyses, within each intervention sequence (i.e., EIAU only, EIAU + LICF, and EIAU + LICF + HICF), we will use the GLME technique to relate repeated measures of sustainment outcomes to time and the following time-varying covariates for each time period: intervention dose, count/frequency of specific implementation techniques, ROSE status and occurrence of critical incidents. The same model will be fit for mediators, and then also for outcomes controlling for hypothesized mediators as time-varying covariates.

Qualitative data will include (1) interviews with clinic respondents to capture variation in sustainment and risk and (2) analysis of implementation process notes. Qualitative interviews will occur every 6 months after baseline (at 6, 12, 18, 24, 30 months) with ~ 60 interviews (clinical and operational respondents at 30 clinics) at each time point. At each time point, clinics will be chosen in the following order: (1) any clinic randomized during the time period, (2) any clinic that changed status (from offering ROSE at adequate fidelity to not, or vice versa), and (3) a few clinics that have sustained well. Deductive codes will be drawn from interview question topics and the Critical Incident Technique specified by Pluye [74] to identify critical incidents and processes related to sustainment. Inductive codes capturing emergent themes will arise from team-level review of the transcripts. Codes will be entered into NVivo. Thematic analysis [75] will be used to identify key themes.

## Discussion

The ROSE Sustainment Study will be among the first randomized trials evaluating the costs and cost-effectiveness (i.e., ROI) of a stepped approach to sustainment, a critical unanswered question in implementation science. Rigor and reproducibility are ensured by the randomized trial design; clear inclusion criteria for participating clinics; manualized protocols and fidelity assessment for EIAU, LICF, and HICF; careful characterization of implementation processes; reliable and valid measures; and transparent power and statistical analyses. The study’s focus on enhancing care of an underserved population, examination of mechanisms and moderators, and ROI analyses increase its relevance. Thus, the study will advance implementation science, knowledge of implementation science mechanisms, and clinical care for at-risk women.

## Abbreviations

EIAU: Enhanced implementation as usual
FQHC: Federally qualified health center
GLME: Generalized linear mixed effects
HICF: High-intensity coaching and feedback
IPT: Interpersonal psychotherapy
LICF: Low-intensity coaching and feedback
MDD: Major depressive disorder
PPD: Postpartum depression
RE-AIM framework: Reach, effectiveness, adoption, implementation, and maintenance together determine public health impact
REP framework: Replicating effective programs
ROI: Return on investment
ROSE program: Reach Out, Stay Strong, Essentials for mothers of newborns
ROSES Study: ROSE Sustainment Study
SMART: Sequential multiple assignment randomized trial
